# Imaging of human papilloma virus associated oropharyngeal squamous cell carcinoma and its impact on diagnosis, prognostication, and response assessment

**DOI:** 10.1259/bjr.20220149

**Published:** 2022-06-23

**Authors:** Philip Touska, Steve Connor

**Affiliations:** 1 Department of Radiology, Guy’s and St. Thomas’ NHS Foundation Trust, London, United Kingdom; 2 Department of Neuroradiology, Kings College Hospital NHS Trust, London, United Kingdom; 3 School of Biomedical Engineering & Imaging Sciences, King’s College London, London, United Kingdom

## Abstract

The clinical behaviour and outcomes of patients with oropharyngeal cancer (OPC) may be dichotomised according to their association with human papilloma virus (HPV) infection. Patients with HPV-associated disease (HPV+OPC) have a distinct demographic profile, clinical phenotype and demonstrate considerably better responses to chemoradiotherapy. This has led to a reappraisal of staging and treatment strategies for HPV+OPC, which are underpinned by radiological data. Structural modalities, such as CT and MRI can provide accurate staging information. These can be combined with ultrasound-guided tissue sampling and functional techniques (such as diffusion-weighted MRI and ^18^F-fludeoxyglucose positron emission tomography-CT) to monitor response to treatment, derive prognostic information, and to identify individuals who might benefit from intensification or deintensification strategies. Furthermore, advanced MRI techniques, such as intravoxel incoherent motion and perfusion MRI as well as application of artificial intelligence and radiomic techniques, have shown promise in treatment response monitoring and prognostication. The following review will consider the contemporary role and knowledge on imaging in HPV+OPC.

## Introduction

Human papilloma virus-associated oropharyngeal squamous cell carcinoma (HPV+OPC) is now recognised as distinct from non-HPV-related squamous cell carcinoma (SCC) of the head and neck (HPV-HNSCC). This is due to its unique epidemiology, histology, clinical behaviour, and outcomes. In particular, HPV+OPC is associated with a significantly improved chemoradiotherapy (CRT) response.^1–4^ and prognosis, with superior 5 year overall survival rates (62–91% and 35–74% for HPV+OPC and HPV-OPC, respectively).^1–4^ Imaging is key to the diagnosis, staging and monitoring of treatment response in OPC and there is increasing recognition of the influence HPV status has on imaging findings.

## Background

### Carcinogenesis

**Figure 1.**
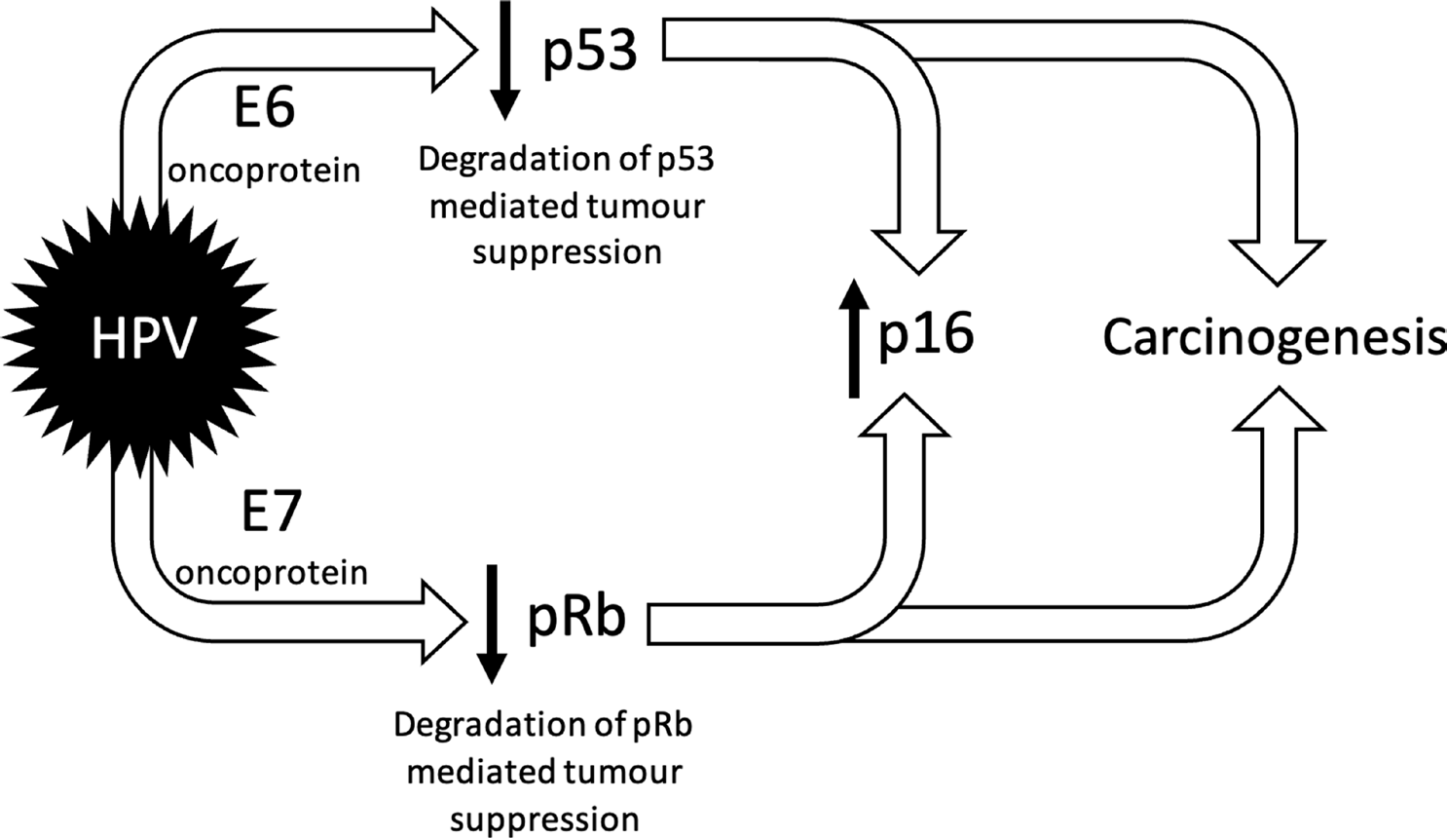
Carcinogenesis in HPV+OPC. Simplified diagram illustrating the effect of HPV oncoproteins E6 and E7, which, once within the host cell, cause degradation of the tumour suppressor protein p53 and retinoblastoma tumour suppression gene product (pRb) respectively. This results in a loss of tumour suppressive abilities, such as arresting the cell cycle and triggering apoptosis and initiation of DNA repair, resulting in carcinogenesis. In addition, there is upregulation of the tumour suppressive p16 protein (that inhibits cyclin-dependent kinase 4A) due to the loss of a negative feedback effect.^8–10^ HPV, human papilloma virus; OPC, oropharyngeal cancer.

HPV+OPC is driven by high risk variants (principally HPV16).^5^ These demonstrate a tropism for oropharyngeal tonsillar crypts, which have a unique structure that is thought to facilitate viral entry into an otherwise immune privileged site.^6,7^ Once the virus has gained access to oropharyngeal tissue, it downregulates tumour suppressive pathways and upregulates p16 transcription (Figure 1).^8,9^

### Histological diagnosis

**Figure 2.**
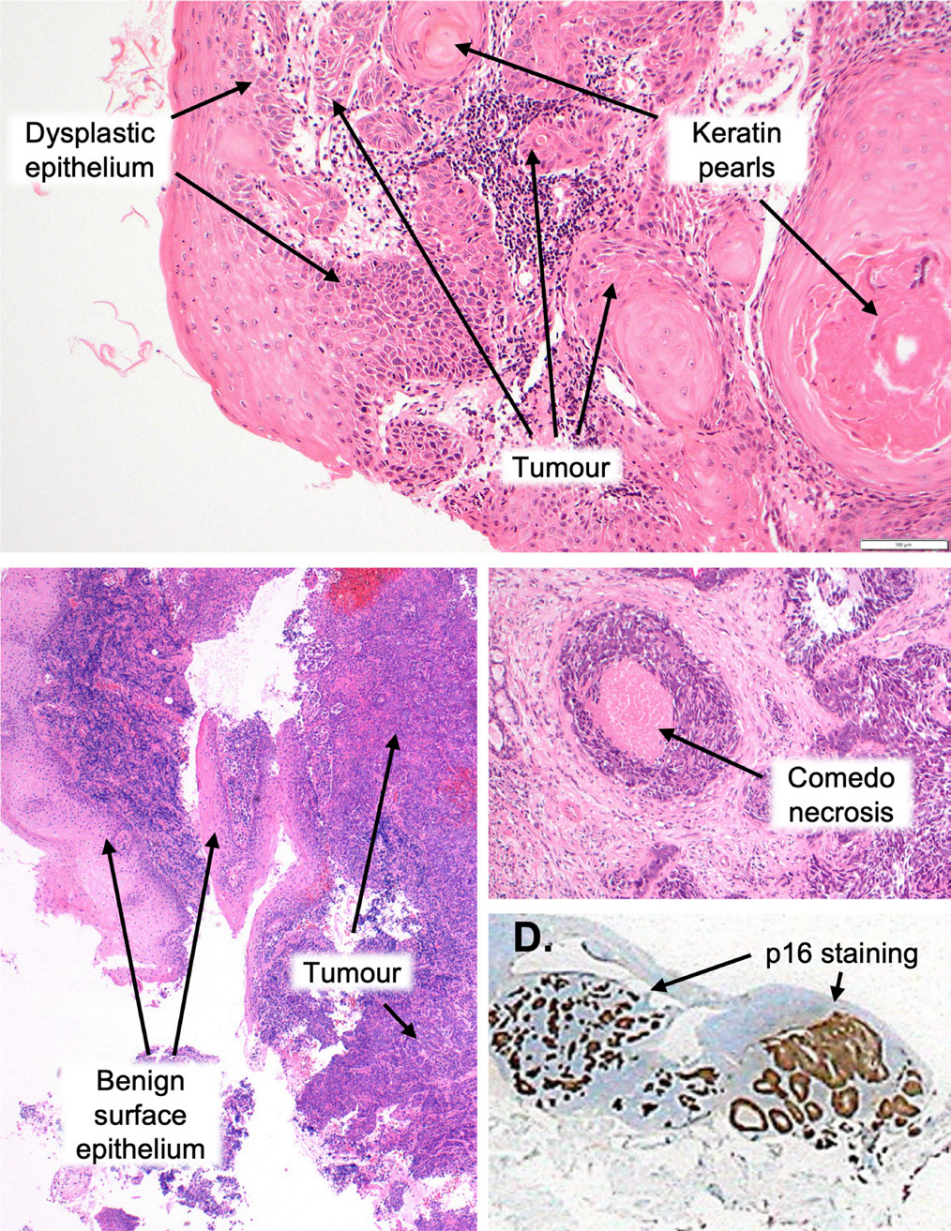
Comparative histology. Low power hematoxilin and eosin-stained slide from the palatine tonsil of a patient with HPV-OPC demonstrating typical histological features (**A**). In particular, HPV-OPC is characterised by progression from dysplasia, keratinisation, an infiltrative growth pattern with angulated nests as well as polygonal cells with abundant mature cytoplasm, distinct cell borders and intracellular bridges. There is also often a strong desmoplastic stromal response.^11,12^ Low power hematoxilin and eosin-stained slides from the palatine tonsil of a patient with HPV+OPC (**B, C**). Typical features of HPV+OPC include a lack of progression from dysplasia, absent keratinisation, an absent desmoplastic response (despite invasive growth), comedo necrosis ( ±cystic degeneration) and lymphoid infiltration is common. The cellular appearance is typified by basaloid cells (including a high nuclear-to-cytplasmic ratio), ovoid spindle shape, hyperchromatic nuclei lacking prominent nucleoli and frequent mitoses.^11,12^ There is also positive staining for p16 (**D**). Histological images courtesy of Dr A. Sandison, consultant head and neck pathologist, Guy’s & St. Thomas’ NHS Foundation Trust. HPV, human papilloma virus; OPC, oropharyngeal cancer.

HPV+and HPV-OPC differ in their microscopic appearances, which can be used to guide diagnosis (Figure 2). In addition, immunohistochemical (IHC) staining for p16 has emerged as an excellent surrogate marker of transcriptionally active HPV infection (with pooled sensitivities and specificities of 94 and 83% respectively).^13–15^ Furthermore, p16 IHC correlates well with HPV *in situ* hybridisation (ISH) techniques, is more cost-effective than detection of HPV oncogene E6 and E7 transcripts and is more specific than HPV DNA polymerase chain reaction (PCR) testing for transcriptionally active HPV infection.^14^ As a result, p16 IHC has been included in the eighth edition of the American Joint Committee on Cancer (AJCC) guidelines as the principle marker of HPV status.^16^ However, in recognition of the fact that p16 positivity is not always HPV driven, many well-resourced centres use a combination of techniques (*e.g.* p16 IHC with ISH or PCR) to increase specificity and prevent false positives.^17^

### Demographics

In contrast to other forms of HNSCC, which are strongly associated with tobacco and alcohol excess, HPV+OPC typically affects younger patients, with or without a smoking history and may relate to certain sexual behaviours.^8,17^ There is also geographic variation, with a higher (and rising) prevalence in high-income Western countries; *e.g.* in the United States and Western Europe, HPV+OPC is thought to account for around 70% of OPC,^8,18,19^ compared to around 22.4% internationally.^20^

### Disease at presentation

Primary neoplasms in HPV+OPC usually arise from the lymphoepithelial tissues of the oropharynx (palatine tonsils and tongue base), but may be very small and therefore clinically occult.^11,21,22^ In contrast, nodal disease in HPV+OPC may be extensive and cystic.^11^ As a result, patients may present with cervical nodal metastases of unknown primary origin, potentially mimicking a second branchial cleft cyst.^21,23^

### HPV+HNSCC at non-oropharyngeal sites

The incidence of HPV+non-oropharyngeal HNSCC is variably reported, but an international histopathological study found 4.4% of oral cancer and 3.5% laryngeal cancers tested positive for HPV markers.^20^

The prognostic significance of HPV+non OPC HNSCC appears to be site dependent; *e.g.* a recent meta-analysis found HPV+laryngeal and hypopharyngeal SCC were associated with improved survival metrics (overall survival hazard ratios of 0.71 and 0.6 respectively), but HPV+ oral cavity SCC was associated with a worse disease-free survival (hazard ratio of 1.81).^24^ In nasopharyngeal SCC, approximately 20.6% are HPV+ and there is an association with non-endemic, Epstein-Barr virus (EBV) negative, lower grade (WHO Grade 1) disease, which is postulated to reflect a distinct subtype.^25,26^ Elsewhere within the upper aerodigestive tract, infection by high-risk HPV33 is associated with the development of HPV-related multiphenotypic sinonasal carcinoma, which typically has a relatively indolent course.^27^

### Staging

HPV+OPC has a greater propensity to present with nodal disease, yet it has a far better prognosis than HPV- OPC. As a result, the eighth edition of the AJCC staging manual includes separate staging for p16+OPC, which has the capacity to downstage the majority of HPV+ OPC cases from Stage IV (using the seventh edition) to stages I or II (using the eighth edition) (Table 1).^16,28^ The changes in the eighth edition predominantly pertain to nodal (N) staging in HPV+ OPC (reflecting the improved prognosis); *e.g.* multiple ipsilateral metastatic lymph nodes are re-classified as N1 rather than N2b disease.^16^ This has an impact on clinical staging groups that can be used to guide treatment (Table 2).

**Table 1.** Comparisons of TNM staging between AJCC seven and AJCC eight for HPV+OPC

	Primary staging				Nodal staging		
	**AJCC 7**		**AJCC 8**		**AJCC 7**		**AJCC 8**
Tis	Carcinoma *in situ*	-	-	N0	No regional metastases	N0	No regional metastases
T1	≤2 cm	T1	≤2 cm	N1	Ipsilateral node≤3 cm	N1	Ipsilateral nodes ≤ 6 cm
T2	>2 cm but ≤4 cm	T2	>2 cm but ≤4 cm	N2a	Single ipsilateral node >3 cm but ≤6 cm maximally	N2	Contralateral/bilateral nodes ≤ 6 cm
T3	>4 cm or extension to lingual surface of epiglottis	T3	>4 cm or extension to lingual surface of epiglottis	N2b	Multiple ipsilateral nodes ≤ 6 cm maximally	N3	Node(s)≥6 cm
T4a	Invades larynx, extrinsic tongue muscles, medial pterygoid, hard palate or mandible (unless tongue base/vallecular primary, in which case lingual epiglottic involvement does not constitute laryngeal invasion)	T4	Invades larynx, extrinsic tongue muscles, medial pterygoid hard palate or mandible/beyond	N2c	Bilateral/contralateral nodes ≤6 cm maximally		
T4b	Invades lateral pterygoid, pterygoid plates, lateral nasopharynx, skull base or encases carotid artery			N3	Node(s) >6 cm maximally		

HPV, human papilloma virus; OPC, oropharyngeal cancer.

Reference: eighth edition of the AJCC cancer staging manual.^16^

**Table 2.** Differences in clinical group staging of HPV+OPC (AJCC seven and AJCC 8)

AJCC 7				AJCC 8			
**T**	**N**	**M**	**Stage**	**T**	**N**	**M**	**Stage**
T1	N0	M0	I	T1-2	N0-1	M0	I
T2	N0	M0	II	T1-2	N2	M0	II
				T3	N1-2	M0	II
T3	N0-1	M0	III	T1-4	N3	M0	III
T1-2	N1	M0	III	T4	N0-3	M0	III
T4a	N0-2c	M0	IVa	Any T	Any N	M1	IV
T4b	N0-3	M0	IVb				
Any T	Any N	M1	IVc				

HPV, human papilloma virus; OPC, oropharyngeal cancer.

Reference: eighth edition of the AJCC cancer staging manual.^16^

### Treatment

Currently, standard treatment for OPC is usually independent of HPV status (Table 3). However, given the markedly better prognosis of HPV+OPC, there has been considerable interest in exploring methods of treatment deintensification so that the deleterious long-term side-effects of CRT^30^ might be reduced without reducing cure rates. Following surgical techniques, such as transoral robotic surgery (TORS) or transoral laser microsurgery (TLM), it may be possible to decrease the adjuvant RT dose or omit concurrent chemotherapy in those without extracapsular spread or positive margins and this is currently being investigated.^31^ Other trials looking at RT dose reduction and treatment modification based on response to induction chemotherapy are currently underway.^32,33^ However, deintensification remains controversial, particularly following the publication of two trials that found reduced survival rates in the groups of HPV+OPC patients treated with a less toxic alternative to cisplatin chemotherapy (cetuximab).^34,35^ In particular, the trials found 2 and 5 year overall survival rates were 97·5 and 84.6% respectively in the cisplatin groups, compared with 89·4 and 77.9% respectively in the cetuximab groups.^34,35^ Application of deintensification arms to the subgroup of HPV+OPC with the lowest clinical risk (limited neck disease and non-smokers) is likely to be important in future trials.^36^

**Table 3.** Management of OPC in the UK

Stage	Management
Early (T1-T2 N0)	Single modality (primary surgery or RT)
Advanced (T3-4 N0/T1-4, N1-N3)	Dual modality (CRT) or triple modality (surgery and adjuvant CRT).

OPC, oropharyngeal cancer.

OPC treatment is dependent on stage.^29^ *RT = radiotherapy, CRT = chemoradiotherapy*.

### Pre-treatment assessment and prediction of HPV+ *vs* HPV- OPC

Given the histological and phenotypic differences between HPV+ and HPV- OPC, and its impact on staging and management, there has been considerable interest in using imaging as a non-invasive means of predicting HPV status prior to pathological diagnosis.

## Structural imaging with CT and MRI

### Primary tumour

**Figure 3.**
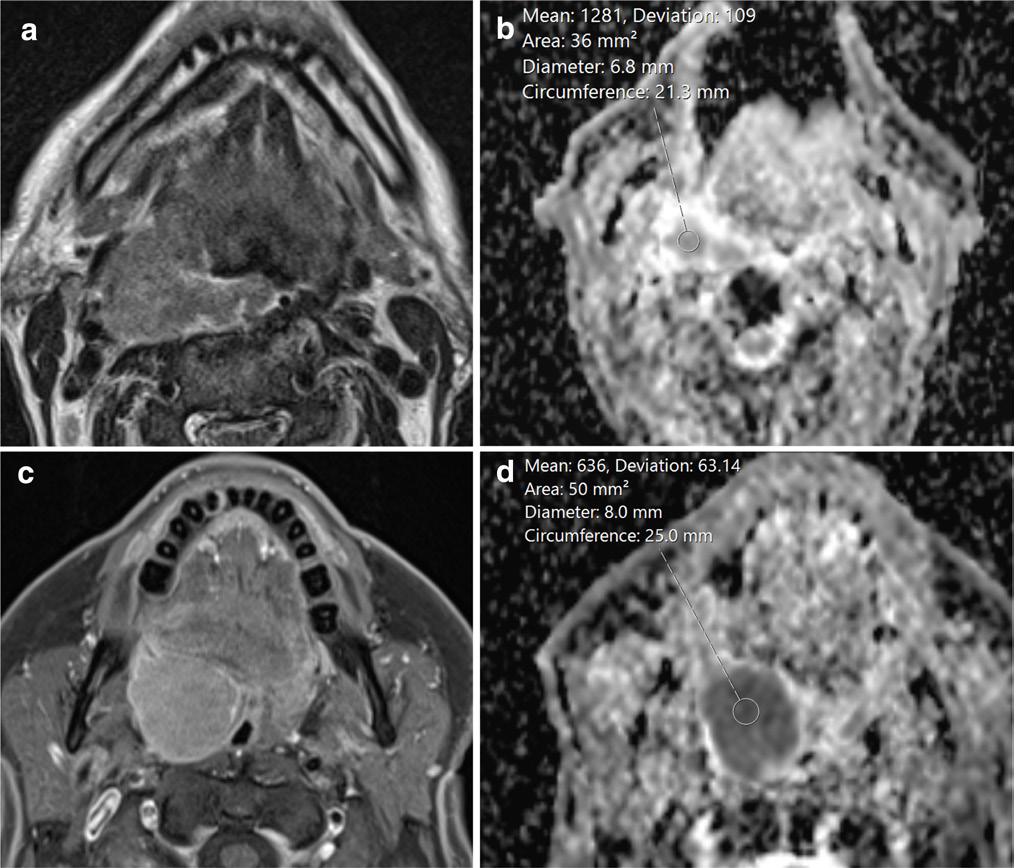
Structural MRI and ADC value differences according to HPV status. Axial *T*_2_W sequence (**A**) demonstrating a bulky HPV- OPC centred on the right palatine tonsil with deep invasion of the styloglossus (arrowhead) ADC map (**B**) in the same patient reveals an intratumoral mean ADC of 1.261 × 10^−3^ mm^2^/s. Axial T1 fat-saturated post-contrast sequence (**C**) demonstrating an HPV+ OPC tumour centred on the right palatine tonsil (arrowheads). It is expansile and markedly exophytic. An ADC map (**D**) through the same tumour reveals a mean ADC of 0.636 × 10^−3^ mm^2^/s. ADC, apparent diffusion coefficient; HPV, human papilloma virus; OPC, oropharyngeal cancer.

Whilst imaging cannot replace histopathology in differentiating HPV+ and HPV- OPC, structural differences in the appearances of the primary tumour have been identified. Cantrell et al reviewed the pre-treatment CT scans of patients with OPC, and found HPV+ primaries were significantly less likely to demonstrate local muscle invasion (6% compared to 26%) and displayed a non-significant tendency towards greater enhancement, exophytic growth and well-defined borders (Figure 3).^37^ Similarly, Chan et al found HPV+ primary tumours (compared to HPV- tumours) were significantly more likely to be smaller (in their anteroposterior and mediolateral axial dimensions) at presentation, exophytic (73% compared to 63%), with well-defined borders (58% compared to 47%), and were significantly less likely to demonstrate ulceration (10% compared to 20%) or necrosis (9% compared to 19%).^38^

### Nodal disease

**Figure 4.**
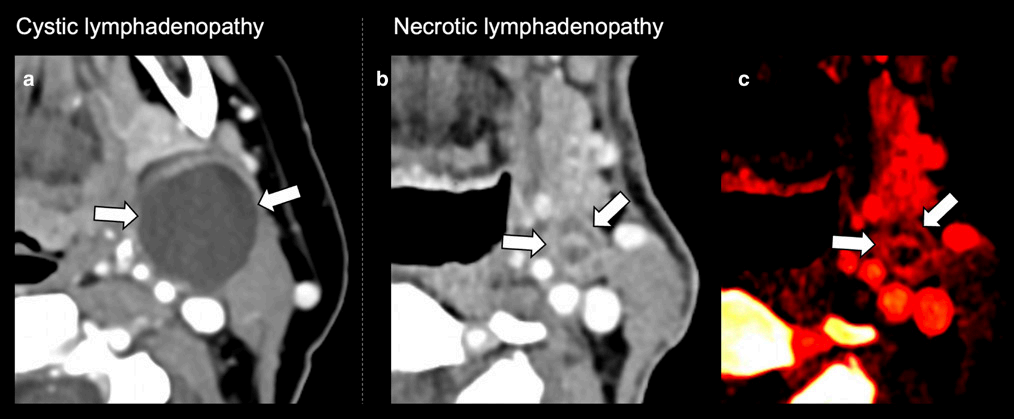
Differentiation of cystic and necrotic cervical lymphadenopathy using cross-sectional imaging. Post- contrast single energy (**A**) and dual-energy (**B, C**) CT images demonstrating nodal features described by Goldenberg et al.^41^ (**A**) Features an example of cystic lymphadenopathy at left level 2A in a patient with HPV+ OPC, demonstrating a round or ovoid mass with a thin (<2 mm) enhancing capsule, homogeneous fluid content, and no internal complex, irregular, or solid area. (**B**) Demonstrates an example of necrotic lymphadenopathy at left level 2A in a patient with HPV- OPC node with thicker solid walls and irregular, complex central low attenuation; these features are accentuated on the iodine map (**C**). HPV, human papilloma virus; OPC, oropharyngeal cancer.

Cystic lymphadenopathy is a recognised feature of HPV+ OPC (possibly relating to the presence of salivary gland type cells or transformation of keratinocytes) and has been proposed as an imaging biomarker.^39,40^ On structural imaging, it can be challenging to differentiate cystic change from necrosis; therefore, Goldenberg et al suggested a series of CT criteria in an effort to aid differentiation (Figure 4).^41^ A subsequent study by Morani et al found cystic change to have a low sensitivity (38.4%), but a higher specificity (73.3%) for HPV+ status in OPC.^39^ The same group also found specificity could be improved (100% compared to 73.3%) by using an additional criterion of low density (<25 Hounsfield units) change within small (<1.5 cm) lymph nodes.^39^ Both Chan et al and Cantrell et al. (using the Goldenberg criteria on CT and MRI respectively) found cystic lymphadenopathy to be significantly more common in HPV+ OPC (36–45%) than HPV- OPC (9–32%).^37,38^ However, there was no relationship between nodal distribution and HPV status.^38^

**Figure 5.**
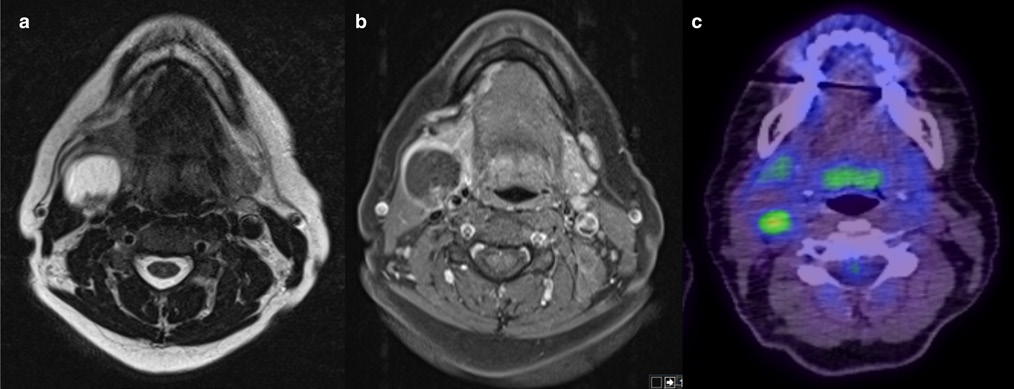
Cystic neck mass evaluation with MRI &^18^F FDG PET-CT. Differentiation between branchial cleft cysts and cystic metastases in HPV+ OPC with an occult primary can be challenging. However, features such as a solid, enhancing mural nodule (seen on axial *T*_2_W image A. and fat-saturated post-contrast Dixon T1 image B or focal uptake on ^18^F-FDG PET-CT (**C**).) are helpful in supporting a diagnosis of malignancy. FDG, fludeoxyglucose; HPV, human papilloma virus; OPC, oropharyngeal cancer; PET, positron emission tomography.

The propensity of patients with HPV+ OPC to develop cystic nodal disease can result in a diagnostic pitfall, particularly when the nodal disease is solitary, and the primary tumour is clinically occult. In such cases, the clinical presentation closely mimics that of a benign second branchial cleft cyst and can lead to malignancy being missed. As a result, there is increasing recognition amongst clinicians and radiologists that adult patients presenting with an apparent second branchial cleft cyst should be suspected of having metastatic OPC until proven otherwise. The risk of malignancy in patients presenting in this way is generally thought to increase with age, notably those over the age of 40 years.^42–45^ As a result, such cases are typically investigated as a carcinoma of unknown primary, including with ^18^F-fludeoxyglucose positron emission tomography-CT, which has been found to have a high negative-predictive value for malignancy of 96% (Figure 5).^46^ MRI is typically also employed to help reveal a clinically occult primary neoplasm and may also be helpful in differentiating branchial cleft cysts from nodal metastases, with the former tending to be larger and lacking septations or features of ENE (Figure 5).^47^

### Ultrasound and ultrasound-guided sampling in the diagnosis of HPV+ OPC

Approximately, 44% of patients with OPC present with a neck mass and ultrasound-guided fine needle aspiration (ultrasound-FNA) is frequently the first-line diagnostic procedure.^48^ However, the sampling adequacy of ultrasound-FNA of cervical lymph nodes may be reduced by the presence of cystic lymphadenopathy in HPV+ OPC, due to paucicellular samples, debris or inflammatory material.^44,49^ Despite this, ultrasound-FNA remains the best initial technique for nodal sampling. Furthermore, the material obtained can be subject to testing for HPV markers (facilitated by cell block preparation).^50,51^ Positive staining can be helpful in differentiating cystic HPV+ OPC metastases from branchial cleft cysts.^52^ Whilst such testing has been shown to correlate well with histological findings, issues remain including a lack of consensus regarding cut-off values for p16 positivity and there is a risk of false negative results (up to 21%), particularly if there is a paucity of material.^50^ There are also issues surrounding obtaining sufficient material for a cell block (up to 48% adequacy in one study).^51^ Ultrasound-guided core biopsy represents an important adjunct to FNA with a higher sensitivity and sample adequacy.^53,54^ Its use may be additive in the setting of non-diagnostic ultrasound-FNA or where HPV testing is required; a meta-analysis found, compared to FNA, core biopsy was able to achieve greater specificity (99% compared to 96%), accuracy (96% compared to 93%), and NPV (95% compared to 90%) in detecting head and neck malignancy.^55^ Nevertheless, this should be balanced with the appreciation that core biopsy is a more traumatic technique with a slightly higher risk profile than FNA, although this may be mitigated with smaller gauge core biopsy needles.

#### Pre-treatment functional and quantitative imaging of HPV+ OPC

### ^18^F-FDG PET-CT and quantitative diffusion-weighted imaging (DWI)

Various imaging techniques can provide functional and quantitative information in patients with HNSCC, but ^18^F-FDG PET-CT and diffusion-weighted imaging (DWI) are the most frequently employed. ^18^F FDG PET-CT yields metabolic information based on glycolytic activity, whilst quantitative DWI with apparent diffusion coefficient (ADC) values provides information on lesion microstructure, such as cellularity.

For ^18^F FDG PET-CT, there is conflicting evidence with respect to its ability to differentiate HPV+ from HPV- OPC primaries, with Tahari et al finding significantly higher glycolytic parameters (including standardised uptake value (SUV)_max_ SUV_peak_ and SUV_mean_) in HPV- OPC, but Clark et al finding no differences in the SUV_max_ between the HPV+ and HPV- groups.^56,57^ In contrast, studies have more consistently demonstrated greater glycolytic parameters in HPV+ OPC nodal metastases.^56–58^ For example, Clark et al found that a nodal SUVmax of >10.8 could reliably predict p16+ disease.^57^

With respect to quantitative DWI, several studies have found lower pre-treatment ADC values in HPV+ OPC primary tumours, which may relate to tumour-infiltrating lymphocyte content.^59–62^ A threshold value of mean ADC for HPV+ OPC has been proposed (<1.027×10^−3^ mm^2^/s, using echoplanar DWI with two B-values (0 and 1000 s/mm^2^) and yielding high sensitivities and specificities (83.33 and 78.57% respectively).^61^ However, the relationship has not been observed in all studies and higher and more variable ADC values have been found in HPV+ OPC, which may relate to intratumoral micronecrosis.^63^ Furthermore, investigation into the relationship between lower ADC and nodal disease in HPV+ OPC has yielded conflicting results..^64,65^ It is important to recognise that the methods of region of interest (ROI) contouring and the incorporation of cystic components is likely to influence the observed ADC values.

### IVIM, perfusion MRI and radiomic analysis of tumour heterogeneity

Whilst not employed in routine clinical practice, advanced imaging and data analysis techniques have been employed in the evaluation of OPC.

Intravoxel incoherent motion (IVIM) MRI is an extension of DWI (using multiple b-values) which accounts for sources of incoherent motion of water molecules within voxels (notably arising from moving blood within capillaries).^66^ As a result, maps reflecting separate diffusion and perfusion components can be created. Lower ADC and tissue diffusion coefficient (D_t_) values have been observed in primary tumours and metastatic lymph nodes of patients with HPV+ OPC, with D_t_ values achieving good levels of sensitivity (85.7–94.9%) and specificity (61.9–64.7%) when combined with risk factors, such as smoking and alcohol intake.^60,67^ However, perfusion-related parameters, including the perfusion fraction (f) and perfusion-related diffusion coefficient (D*), have not been found to be discriminatory.^67^ Due to the paucity of data and time consuming nature of the IVIM sequence, its use remains primarily in the research domain.

Dynamic contrast-enhanced (DCE) MRI involves rapid imaging of a bolus of contrast passing through a volume of tissue using 3D spoiled gradient echo sequences.^68^ The approximately linear relationship between gadolinium concentration and T1 signal can be used to derive perfusion and permeability metrics that are thought to reflect the tumoral microenvironment.^69^ However, its role in predicting HPV status is unclear. In particular, one study found the DCE MRI metrics volume transfer constant (K_trans_) and flux rate constant (k_ep_) (reflecting capillary permeability) were significantly lower in HPV- and epidermal growth factor receptor (EFGR)+ oral cavity SCC and OPC and a K_trans_ cut-off value of 0.18 min^−1^ could be used to predict HPV+ status with a sensitivity of 80% and specificity of 85.7%.^70^ However, a more recent study found no difference in DCE parameters between HPV+ and HPV- OPC.^60^ Given the inconsistencies, further data are required before it can be relied upon to aid prediction of HPV status.

Radiomics involves the extraction of quantitative data from imaging studies that cannot be appreciated by the human eye and can be facilitated by artificial intelligence (AI) techniques. Radiomics-based phenotyping (using 6 of 170 radiomic features) derived from segmented contrast-enhanced MRI studies has shown promise in differentiating HPV+ from HPV- OPC on MRI (with an area under the receiver operator characteristic curve of 0.982 on a training data set and 0.744 on the test data set).^71^ Furthermore, textural histogram parameters have been found to differ significantly, depending on HPV status on pre-treatment CT studies, with Lee et al finding the entropy parameter could be used to predict priary tumour HPV status with an accuracy of 80% in the training and 75% in the validation data sets.^72–74^ Additionally, evaluation of ADC-derived histograms for HPV- oral cavity SCC and OPC have been found to display a more normal distribution (compatible with greater histological heterogeneity) as compared to HPV+ oral cavity SCC and OPC, which is characterised by greater kurtosis and skewness (thought to reflect more homogenous histological features).^75^ Despite promising data, the use of radiomics remains at an early stage with persistent challenges pertaining to reproducibility and generalisability, which limit its use to the research domain.^76^

### Prediction of treatment response

Whilst HPV+ OPC has an excellent prognosis, there remains significant interest in identifying imaging biomarkers that could help identify tumours which are less likely to respond to CRT. This would help guide more proactive surveillance and to prompt more intensive or modified treatment plans such as radiotherapy dose escalation or surgery.

## Pre-treatment structural imaging with CT and MRI

### Imaging of primary tumour to predict complete surgical resection

TORS and TLM can be employed as part of a treatment deintensification strategy in HPV+ OPC; however, discovery of prognostically unfavourable histological features (*e.g.* positive surgical margins at the primary site) are an indication for adjuvant CRT. In such cases, the intended deintensification benefits of surgery are lost and patients undergo triple (rather than double) modality therapy.^77^ Therefore, predicting which cases are likely to have surgically unfavourable features pre-operatively using imaging is of clear benefit. MRI has been shown to reliably predict positive surgical margins (with a sensitivity of 87.5% and specificity of 92.3%) based on pharyngeal constrictor muscle obscuration or invasion on MRI.^78^ MRI may also be used to identify invasion of other key muscular structures, such as the extrinsic tongue muscles, pterygoid muscles and eustachian tube or high risk anatomical variants, such as a retropharyngeal internal carotid artery that might contraindicate TORS.^79^

### Imaging of nodal disease to predict treatment outcome

Cystic metastases have been found to be associated with an improved prognosis in studies of HNSCC, but this finding can be accounted for by the confounding inclusion of patients with HPV+ OPC.^80^ However, even amongst patients with HPV+ OPC, cystic metastases may have a prognostic significance; in particular the presence of solid (as opposed to cystic) nodal metastases have been found to correlate with a higher risk of treatment failure and poorer disease-free survival (DFS) in HPV+ OPC (hazard ratio of 2.21).^81^ In addition, the nodal distribution may be important since the presence of retropharyngeal lymphadenopathy (coupled with a more advanced stage) has been associated with a reduced time to distant metastatic failure in 266 patients with HPV+ OPC (combined C-index of 0.84).^82^

ENE is known to be an indicator of poor prognosis in HPV- HNSCC (with higher rates of recurrence and distant metastasis).^16^ As a result, an additional (N3b) stage was added for HPV- HNSCC (including HPV- OPC) in the eighth edition of the AJCC staging manual.^16^ Since early data were unclear, ENE was not included as a separate nodal stage in the eighth edition of the AJCC manual for HPV+ OPC.^16^ However, more recent evidence suggests a correlation between ENE and poor outcomes in HPV+ OPC treated surgically, with An et al finding ENE was associated with worse 3 year OS (89.3% compared to 93.6%; *p* = .01), and Bauer et al finding ENE-positivity was associated with a hazard of death of 1.90; 95% CI: 1.35–2.67) compared to ENE-negative cases.^83–85^ The clinical definition of ENE in the latest AJCC staging manual is based upon physical examination rather than radiological findings, but structural imaging has been used to predict ENE based on a number of criteria (Table 4). A recent meta-analysis found CT and MRI, to have pooled sensitivities of 73 and 60% and pooled specificities of 83 and 96% for identifying ENE in HNSCC, with differences between the modalities being non-significant.^77^ The same authors found imaging to be less reliable in HPV+ OPC, with a significantly lower specificity in HPV+ OPC (74%) as compared to HPV- oral cavity or all HNSCC (87%) and postulated that the lower reliability might be due to the inclusion of central necrosis in the diagnostic criteria.^77^ As a result, it remains prudent to exercise caution when predicting ENE on imaging in cases of HPV+ OPC.

**Table 4.** Radiological features of ENE

ENE features with pooled statistical data	
*Infiltration of adjacent planes†*	Pooled sensitivity: 51% (95% CI, 34–67%) Pooled specificity: 94% (95% CI, 76–99%)
*Central necrosis*	Pooled sensitivity: 81% (95% CI, 67–91%) Pooled specificity: 65% (95% CI, 57–72%)
**Additional ENE features lacking pooled statistical data**	
*Indistinct/ill-defined margins* *Lobulated contours* *Perinodal fat stranding* *Nodal matting*	

Of note, infiltration of adjacent fat planes has shown to have lower interobserver agreement (correlation coefficient of 0.46) compared to central node necrosis (correlation coefficient of 0.79).ENE, extranodal extension; Source: Meta analysis by Park et al.

### Pre-treatment functional and quantitative imaging biomarkers

^18^F FDG PET-CT has been found to outperform CT in predicting time to locoregional recurrence based on SUV metrics [metabolic tumour volume (MTV), total lesion glycolysis (TLG) and SUV_2.5_in HPV+ OPC.^82^ Furthermore, several studies have found poorer outcomes to be associated with elevated glycolytic parameters, including higher tumoral and/or nodal SUV_max_, MTV and TLG levels.^86–91^ However, some conflicting data exist, including one study that found elevated TLG (>135.3 g) to be associated with favourable survival statistics (particularly for HPV OPC).^92^ It is possible that AI techniques could aid risk stratification following pre-treatment ^18^F FDG PET-CT in future.^93^

Evaluation of pre-treatment ADC values in patients with HNSCC has revealed an association between higher ADC values and poorer post-CRT outcomes.^94–99^ It is proposed that this relates to histological findings of higher stromal content, lower cellularity and micronecrosis, all of which are known to result in greater treatment resistance.^65^ Such studies also demonstrated an association between lower pre-treatment ADC values and favourable post-treatment survival outcomes in OPC and other HNSCC; however, until recently, little data were provided on HPV status.^94,96,98,99^ It has since been demonstrated that the association between low pre-treatment ADC values and improved outcomes is largely lost when the confounding factor of HPV+ OPC (characterised by lower ADC values) is corrected for.^62,65,88,100–102^ For example, Cao et al found HPV- OPC primary tumours had significantly greater pre-treatment ADC_mean_ (1.48 µm^2^/ms) compared to HPV+ OPC primary tumours (ADC_mean_ of 1.34 µm^2^/ms), but this was not replicated in metastatic lymph nodes.^102^

Radiomic techniques have been applied to prognostication with promising results. For example, Song et al developed radiomic risk score classifiers for patients with HPV+ and HPV- OPC based on pre-segmented CT studies and found that radiomic signatures generated from the data could be used to predict HPV status (accuracy of 76%) and DFS (hazard ratio of 1.97).^103^ Bogowicz et al found an association between a 3-feature radiomic signature indicative of a more heterogenous CT density distribution and poorer local control (with a concordance index of 0.75 for the training cohort and 0.78 for the validation cohort).^104^ However, as with prediction of HPV status, there remains limited reliable evidence for radiomics in HNSCC prognostication, with a dearth of validation and reproducibility studies.^105^

It is worth noting that a degree of caution is required when interpreting quantitative data and thresholds derived from medical imaging, since differing methodologies, patient cohorts and analysis techniques can influence values obtained.

### Intra- and early post-treatment evaluation

Imaging following the initiation of treatment for HPV+ OPC has been performed in clinical studies to enable adaptive radiotherapy techniques such as rapid intensification (*e.g.* dose painting) or deintensification of treatment. Alternatively, it may be performed post-treatment, in order to detect early residual or recurrent disease, since timely intervention with salvage surgery is required before locoregional disease becomes irresectable. In the latter setting, there is an intrinsic trade-off between imaging early enough to detect remediable treatment failure and imaging late enough for treatment-related tumour shrinkage to manifest, so that failure is not declared prematurely.

### Intratreatment quantitative imaging

Since a reduction in tumour dimensions may be delayed, evaluation of intratreatment imaging response has focused on quantitative techniques DWI, IVIM and DCE.^106^ For HNSCC in general, favourable responses to treatment are characterised by greater percentage increases in ADC (using DWI and IVIM) and D (using IVIM) values as well as more rapid tumoral regression.^63,107–112^ Conversely, lower intratreatment ADC and higher pre- and intratreatment D values, as well as larger reductions in f, have been associated with regional treatment failure.^112–114^ For example, Marzi et al found that patients with regional failure had higher mid-radiotherapy D values (1.28 × 10^−3^mm^2^/s compared to 1.09 × 10^−3^mm^2^/s) as well as reductions in f and D*×f from the baseline.^113^ In patients with HPV+ OPC, Ding et al found complete response to treatment correlated with lower pre-treatment ADC and D values and relatively greater changes in mid-treatment ADC, D and f (characterised by rises in these values), achieving an AUC of 0.87, sensitivity of 0.63, specificity of 0.85 and accuracy of 0.78.^115^ DCE MRI has been applied to a mixed (p16+ and p16-) cohort in the context of a multiparametric approach, finding that persistently low blood volume derived from DCE data is associated with a higher risk of distant failure.^102^

^18^F FDG PET-CT, studies carried out mid treatment (*e.g.* 3 weeks following commencement) have also been found to be of utility in identifying patients at risk of treatment failure in HNSCC.^116–119^ For HPV+ OPC, reductions in nodal metabolic tumour volume on mid-treatment scans have been identify a subgroup of patients at low risk of locoregional-failure but higher risk of distant-metastatic-failure.^120^

## Early post-treatment imaging

With respect to early post-treatment imaging of OPC, both qualitative and quantitative metrics can be evaluated, but the choice of modality and timing of imaging are important considerations.

### Qualitative and semi-quantitative assessment of treatment response

MRI is complementary to ^18^F FDG PET-CT in the post-CRT setting and offers advantages over CT due to superior contrast resolution.^121^ In particular, well-described features visible on conventional anatomical MRI sequences can aid differentiation of recurrence from treatment-related change (Table 5).^122,123^

**Table 5.** MRI features associated disease recurrence *vs* early or late treatment-related change

	Disease recurrence	Inflammatory oedema	Late fibrosis
*Shape*	Mass-like	Diffuse	Linear/triangular
*Signal on T_2_WI*	Intermediate	High	Low
*Enhancement*	Moderate	Avid	Minimal/absent

Adapted from Ailianou et al.^122–125^

At the primary site, measured reductions in tumour size on MRI are important in confirming response.^126^ However, measurement may be challenging in the setting of small or inconspicuous HPV+ OPC primaries. For nodal disease in HNSCC, MRI-based measurements of reductions in the size and volume of the solid components have been shown to be of greater importance for detecting residual disease than necrosis or ENE.^127,128^ Volume measurements of the primary tumour may also be helpful, with an association between higher post-CRT tumour volumes (both during and 6 weeks after therapy) and local failure.^129^ Relative to CT, MRI is particularly helpful in this situation, given its superior ability to differentiate between tumour and peritumoral oedema^130^ Both CT and MRI may be used to evaluate post-treatment changes in nodal volume, with greater percentage reductions associated with treatment success.^127,128^

The optimum timing of post-CRT MRI has recently been examined by Connor et al. In this study, nodal response to therapy (as measured on axial sequences) in HPV- OPC was most significant at 6 weeks following completion of CRT and had changed little by 12 weeks. Conversely, in HPV+ OPC, there was significant continued reduction in nodal size between the 6 and 12 week scans. In view of this, earlier post-CRT MRI may be appropriate for HPV- OPC, but 12 week scanning remains appropriate for HPV+ cases with nodal disease.^130^

**Figure 6.**
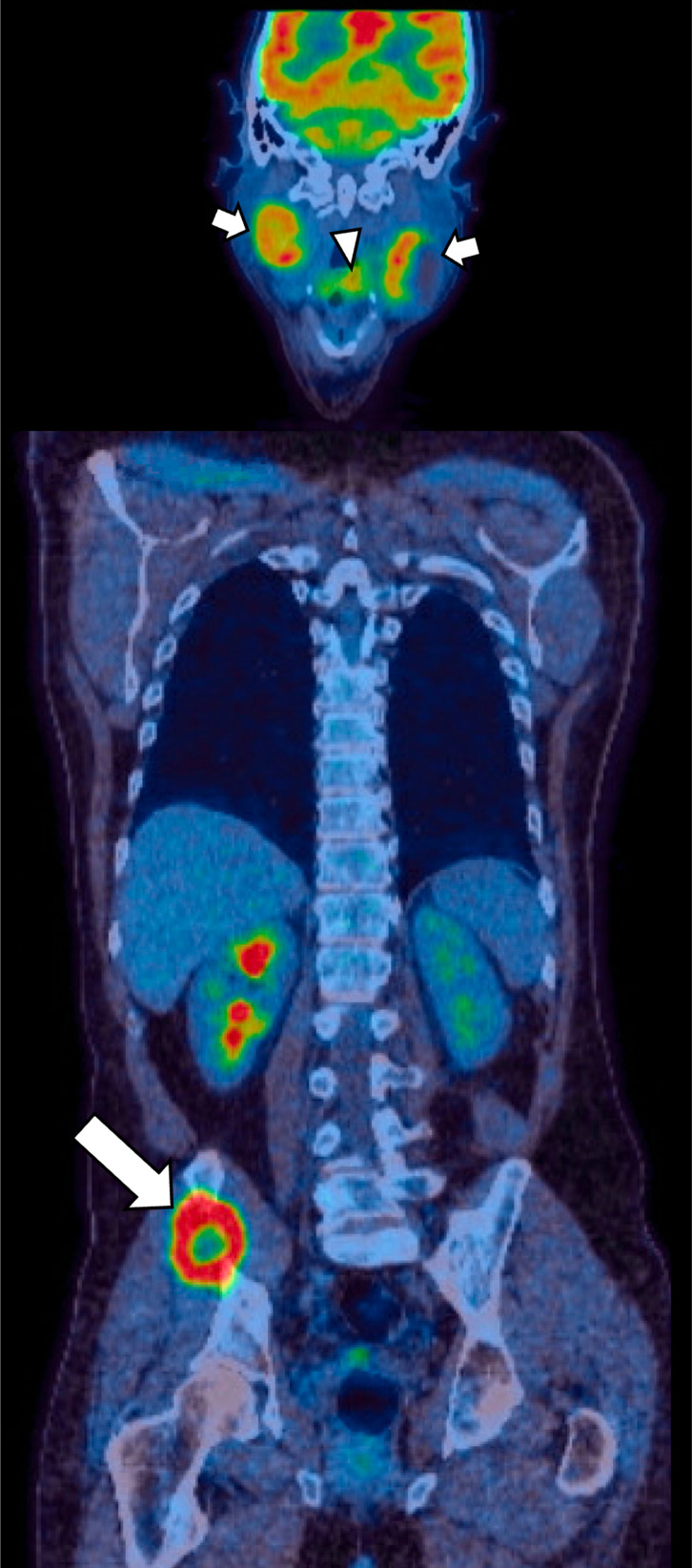
Use of ^18^F FDG PET-CT in the detection of distant metastases. A pre-treatment ^18^F FDG PET-CT in a patient with HPV+ OPC reveals a left tongue base primary (arrowhead), with bilateral partially cystic cervical nodal metastases (small arrows) and a right iliac bone metastasis (large arrow). The latter was histologically confirmed as SCC staining positively for p16 on IHC. FDG, fludeoxyglucose; HPV, human papilloma virus; IHC, immunohistochemical; OPC, oropharyngeal cancer; PET, positron emission tomography; SCC, squamous cell carcinoma.

Metabolic imaging with ^18^F FDG PET-CT is widely used for the identification of treatment failure following CRT for Stage III and IV OPC and other HNSCC, since structural imaging is often difficult to interpret in the setting of treatment related anatomical distortion.^131,132^ It is particularly helpful for ruling out disease owing to excellent negative-predictive values (which typically exceed 90%)..^18,133–139^ It is also capable of detecting distant metastatic disease (Figure 6) which may be relevant to HPV+ OPC following treatment, since it is associated with a higher rate of lung metastases and propensity to involve unexpected anatomical locations.^140^

Post-CRT ^18^F-FDG PET-CT may be carried out 12 weeks following completion of treatment.^141^ However, false positives (due to elevated glycolysis caused by muscle uptake, treatment-related or other non-malignant inflammatory processes) remain problematic and can lead to unnecessary invasive procedures.^142,143^ This is compounded in the setting of HPV+ OPC where there is evidence to suggest that the positive predictive value of a 12 week post-CRT ^18^F FDG PET-CT is particularly low (30% in one study).^144^ Furthermore, nodal lesions in HPV+ OPC have been shown to demonstrate a different pattern of involution, with a more rapid initial decrease in size followed by a longer time to a complete resolution (particularly for cystic lymphadenopathy).^145–147^ This may be addressed by serial or later ^18^F FDG PET-CT scanning. There is now a trend for ^18^F-FDG PET-CT to be performed at a later time interval following HPV OPC treatment, and a study by Liu et al, the number of false positives were reduced by carrying out a repeat ^18^F FDG PET-CT (at 16 weeks) in cases of incomplete nodal response at 12 weeks.^148^

### Quantitative assessment of treatment response

Post-treatment ^18^F FDG PET-CT metrics have been used to predict survival and recurrence in HNSCC. In general, higher levels of SUV_max_ following treatment and a reduced change relative to the pre-operative SUV_max_ are indicators of poor prognosis.^116,137,149–153^ MTV has also been utilised in HNSCC, with Murphy et al finding a larger post-radiation volume being associated with a poorer prognosis.^154^ Some authors have identified cut-off values in patients with HPV+ OPC, but there is variation in the literature. For example, Vainshtein et al used post-treatment SUV_max_ thresholds of 6.5 for the primary tumour and 2.8 for the neck to define complete response, with a high NPV (91–98%), albeit with a low sensitivity and PPV (0–33%).^155^ Chan et al used a lower SUV_max_ threshold of 2, which was associated with a high NPV of 91.7% but lower sensitivity (75% and PPV of 37.5%).^138^ There is evidence that ^18^F FDG PET-CT has a lower PPV in HPV+ HNSCC (20%) compared to HPV- disease (62.5%).^137^ This may be due to the unique response of HPV+ OPC to treatment, with an increased cytotoxic T-cell-based response (resulting in metabolically active inflammation) and greater radiosensitivity (resulting in smaller tumour residua that take longer to regrow and become detectable).^156^

DWI and ADC maps can be used as a surrogate marker of microstructural treatment-related tumoral changes. In particular, therapy-induced tumour necrosis is thought to lead to an increase in diffusivity and therefore ADC values.^130^ Post-CRT increases in ADC metrics following CRT have been associated with disease-free survival in HNSCC; in particular, a rise in post-treatment ADC as well as an increased change in ADC relative to baseline correlated with a favourable prognosis.^97,111,157,158^ For example, Connor et al found HNSCC patients with higher post-treatment ADC_mean_ values 6 and 12 weeks following RT were associated with increased 2 year disease free survival rates.^158^ At 6 weeks, an optimum threshold of 1405 10^–6^ mm^2^/s was derived (with a sensitivity of 83%, specificity of 80%, PPV of 39% and NPV of 97%) and at 12 weeks an optimum threshold of 1840 10^–6^ mm^2^/s was derived (with a sensitivity of 83%, specificity of 57%, PPV of 22% and NPV of 96%).^158^

However, interpretation alongside anatomical MRI sequences is imperative since treatment-related scarring may also produce low ADC values.^159^

### Ultrasound and ultrasound-guided sampling in the follow-up of HPV+ OPC

**Figure 7.**
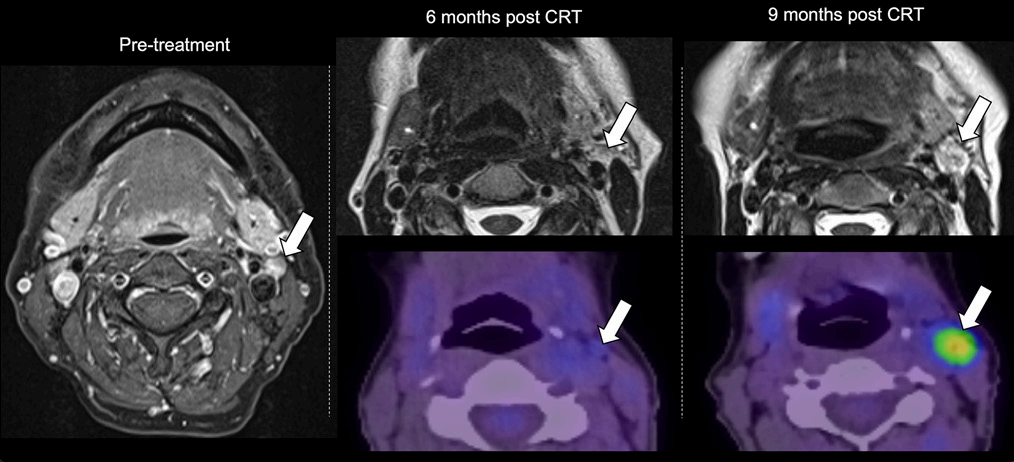
Follow-up of cervical metastatic disease. Far left: Axial fat-saturated post-contrast T1W sequence through the neck of a patient with HPV+ OPC prior to treatment demonstrating a small, but pathological, left level II metastatic node (arrow). Middle panel: the upper image demonstrates slight reduction in the size of the left level II node on a *T*_2_W MRI sequence at 6 months following CRT, but it has not completely regressed. The lower image is from a contemporaneous ^18^F-FDG PET-CT which demonstrates a lack of significant uptake at this location. Right panel: the upper *T*_2_W MRI sequence at 9 months following CRT shows that the left level II nodal deposit has increased in size (arrow) and a contemporaneous ^18^F-FDG PET-CT demonstrates increased uptake. Recurrent disease was confirmed on biopsy. FDG, fludeoxyglucose; HPV, human papilloma virus; IHC, immunohistochemical; OPC, oropharyngeal cancer; PET, positron emission tomography; SCC, squamous cell carcinoma.

At the time of initial post-CRT evaluation, a subset of patients will be deemed to have neither completely responded nor progressed. For example, there may be partial metabolic response on ^18^F FDG PET-CT or there may be visible residua on CT or MRI with reduced ^18^F-FDG uptake (Figure 7).^160^ Ultrasound-guided FNA has a role in evaluating such equivocal nodal findings; however, there are impediments to its utility due to treatment-related fibrosis and necrosis, which can make evaluation of greyscale appearances challenging and limit the yield of diagnostic material.^161^ In patients treated with CRT for HNSCC, there are reports of high false positive and false negative rates for ultrasound-FNA^160,162,163^ In the context of HPV+ OPC, there is a further challenge with regard to the longer time for complete regression of nodal lesions, which may be compounded by challenges interpreting the viability of irradiated cells on FNA cytology specimens, leading to reduced reliability.^160^ Therefore, serial ultrasound demonstrating regression can be helpful in guiding management.^145^ Overall, ultrasound-FNA is a useful tool for evaluating the post-treatment neck, but its limitations should be considered and results interpreted in a multidisciplinary setting and in the context of other clinicoradiological parameters.

### HPV antibodies as tumour markers

Although still at an early stage, serological assays capable of detecting HPV-related antibodies (*e.g.* HPV16 E6 and HPV16-L1 DRH1) within blood specimens have shown early promise in prognosis and post-treatment surveillance.^164,165^ Such assays may provide complementary data and increase the specificity of imaging findings.

## Conclusion

HPV+ OPC has emerged as a distinct clinical entity and an understanding of HPV+ OPC. Whilst identification of HPV status through tissue sampling remains the gold standard, pre-treatment imaging features may enable early identification to refine diagnostic and therapeutic approaches, whilst recognition of the expected post-treatment evolution will help guide investigation for residual disease. Moreover, appreciating the impact of HPV+ OPC on imaging biomarkers will allow their appropriate application for prognostication and treatment monitoring.
